# Insurance type and sepsis-associated hospitalizations and sepsis-associated mortality among US adults: A retrospective cohort study

**DOI:** 10.1186/cc10243

**Published:** 2011-05-23

**Authors:** James M O'Brien, Bo Lu, Naeem A Ali, Deborah A Levine, Scott K Aberegg, Stanley Lemeshow

**Affiliations:** 1 Department of Internal Medicine, Division of Pulmonary, Allergy, Critical Care and Sleep Medicine, Center for Critical Care, The Ohio State University Medical Center, 201 Davis HLRI, Columbus, OH 43221, USA; 2 College of Public Health, The Ohio State University, 320 West 10th Avenue, B-110 Starling Loving Hall, Columbus, OH 43221, USA; 3 Department of Medicine, Division of General Medicine, University of Michigan, 300 North Ingalls, 7C27, Ann Arbor, MI 48109, USA

## Abstract

**Introduction:**

Socio-demographic and clinical factors associated with increased sepsis risk, including older age, non-white race and specific co-morbidities, are more common among patients with Medicare or Medicaid or no health insurance. We hypothesized that patients with Medicare and/or Medicaid or without health insurance have a higher risk of sepsis-associated hospitalization or sepsis-associated death than those with private health insurance.

**Methods:**

We performed a retrospective cohort study of records from the 2003 Nationwide Inpatient Sample. We stratified the study cohort by Medicare age-qualification (18 to 64 and 65+ years old). We examined the association between insurance category and sepsis diagnosis and death among admissions involving sepsis. We used validated diagnostic codes to determine the presence of sepsis, co-morbidities and organ dysfunction and to provide risk-adjustment.

**Results:**

Among patients 18 to 64 years old, those with Medicaid (adjusted odds ratio (AOR) 1.50), Medicare (AOR 1.96), Medicaid + Medicare (AOR 2.22) and the uninsured (AOR 1.18) had significantly higher risk-adjusted odds of a sepsis-associated admission than those with private insurance (all *P *< 0.0001). Those with Medicaid (AOR 1.17, *P *< 0.001) and those without insurance (AOR 1.45, *P *< 0.001) also had significantly higher adjusted odds of sepsis-associated hospital mortality than those with private insurance. Among those 65+ years old, those with Medicaid (AOR 1.43), Medicare alone (AOR 1.13) or Medicaid + Medicare (AOR 1.62) had significantly higher risk-adjusted odds of sepsis-associated admission than those with private insurance and Medicare (all *P *< 0.0001). Among sepsis patients 65+, uninsured patients had significantly higher risk-adjusted odds (AOR 1.45, *P *= 0.0048) and those with Medicare alone had significantly lower risk-adjusted odds (AOR 0.92, *P *= 0.0072) of hospital mortality than those with private insurance and Medicare. Lack of health insurance remained associated with sepsis-associated mortality after stratification of hospitals into quartiles based on rates of sepsis-associated admissions or mortality in both age strata.

**Conclusions:**

Risks of sepsis-associated hospitalization and sepsis-associated death vary by insurance. These increased risks were not fully explained by the available socio-demographic factors, co-morbidities or hospital rates of sepsis-related admissions or deaths.

## Introduction

Sepsis is a common cause of hospitalization and intensive care unit admission. In 1995, there were approximately 750,000 cases of sepsis in the United States (US) with a 30% mortality rate during hospitalization, resulting in 215,000 deaths annually [1]. Costs of sepsis-related hospitalizations were considerable with direct costs of $16 billion [1]. These estimates did not include the costs of post-hospital care, of lost employment or of informal care-giver assistance. Because of the numbers of people affected and costs involved, sepsis is a condition which warrants attention from insurers as a target to improve outcomes for their enrollees and to contain health care costs.

Some risk factors for sepsis and sepsis-related mortality, including older age, non-white race, and specific co-morbidities, are more common among patients with Medicare and/or Medicaid or no health insurance [2,3]. Differences in insurance coverage may also be associated with risk of sepsis or sepsis-related mortality because of differences in access to care, disparities in provided care, overall health status or other unknown factors. An association between insurance coverage and sepsis may provide incentive for payers to target sepsis as a disease for organized intervention, such as value-based purchasing utilizing performance measures, such as time-to-antibiotic administration for septic shock patients [4]. Furthermore, an association between insurance coverage and sepsis, which is independent of known risk factors, would call attention to these disparities in risk for sepsis and poorer outcomes from sepsis among those without private insurance and exploration of the mechanism of such a relationship to identify modifiable factors.

Using nationally representative hospital-based data, we assessed the association between health insurance type and sepsis-related hospitalizations and sepsis-related mortality. We hypothesized that those with Medicare and/or Medicaid and those without health insurance would have higher adjusted odds of sepsis and sepsis-related death than patients with private health insurance.

## Materials and methods

### Ethics statement

Because the Nationwide Inpatient Sample does not include any patient identifiers, the Ohio State University Institutional Review Board waived the requirement of review and consent.

### Data source

This study utilized data from the 2003 version of the Nationwide Inpatient Sample (NIS) [5], the largest all-payer inpatient care database in the US. It contains data which approximate a 20% stratified sample of US hospitals, including private, public and academic hospitals. Weights are provided for each year of the database to allow for calculation of national estimates of all hospitalizations. We included only adult records (≥18 years old) with payer information. To reduce the likelihood of a single hospitalization appearing in the study multiple times and transfer bias [6], we excluded records whose admission source was listed as "another hospital" and those with a discharge status of "transfer to a short term hospital".

### Definitions

Because Medicare has an age-specific qualification, we stratified analyses by patient age (18 to 64 and 65+ years old). The insurance category was recoded from the primary and secondary payers abstracted from the NIS record, which was provided by state-specific sources [7]. Because of variations by state in coding procedures, we excluded records with either primary or secondary payers coded as "no charge" (approximately 0.3% of all records in the database) or "other" (3.2% of records in the database). For the younger age-stratum (18 to 64 years old), records with Medicaid and Medicare listed as primary and secondary payer (or vice versa) were categorized as "Medicaid + Medicare." The remaining records with Medicare as a primary or secondary payer were categorized as "Medicare," regardless if they had additional private insurance. Finally, remaining records were classified as "Private Insurance", "Medicaid" or "Uninsured" as appropriate. For the older age-stratum (65+ years old), "Medicaid + Medicare" was categorized as for the younger age stratum. Remaining records with Medicare as a payer were categorized as either "Medicare alone" or "Private Insurance plus Medicare" if there was no additional listed payer or if commercial insurance was also listed, respectively. Remaining patients were categorized as "Medicaid" or "Uninsured" as appropriate. The reference group was "Private Insurance" for the younger age-stratum and "Private Insurance plus Medicare" for the older age stratum.

For the analyses examining sepsis-associated hospitalization, we based the diagnosis of sepsis upon validated ICD-9 codes [8], namely, if the discharge record contained one or more codes for sepsis (038 (septicemia), 020.0 (septicemic), 790.7 (bacteremia), 117.9 (disseminated fungal infection), 112.5 (disseminated candida infection), and 112.81 (disseminated fungal endocarditis) as a primary or secondary diagnosis. Organ dysfunction was defined as the presence of previously validated and utilized ICD-9 and/or Current Procedural Terminology Codes (CPT) [8,9]. For the analyses examining sepsis-associated mortality, we analyzed only those records with a qualifying sepsis ICD-9 and considered the patient to have died in the hospital if the discharge disposition indicated the patient had died.

### Statistical analyses

We generated descriptive statistics regarding the number of admissions and those associated with sepsis, severe sepsis and sepsis-associated deaths. We also report length of stay for these admissions.

Because age is a qualifying criterion for Medicare eligibility and is also associated with sepsis risk, we performed analyses by age-strata (18 to 64 years and 65+ years). First, we estimated the unadjusted association between insurance category and sepsis within each age-stratum. We used multivariable logistic regression to adjust for known risk factors for sepsis, including demographic information [8,10]) and conditions associated with increased sepsis risk [9,11-15], based on ICD-9 and/or CPT codes. In instances in which patient race was absent (approximately 25% of records), we recoded race as "missing" and included the record in analyses. We also included a co-morbidity index (Charlson-Deyo score [16]), categorized based on preliminary analysis determining best fit with the odds of sepsis. Because individual hospitals contribute multiple records, design-based adjustments are used to provide valid estimates accounting for the correlation among records from the same hospital. Hospitals were identified as clusters in all analyses, as recommended in the HCUP method report [17].

For the analyses of sepsis-associated death, we included only sepsis-associated admissions and considered hospital mortality the dependent variable. Unadjusted odds were estimated between insurance category and mortality by age stratum. We constructed a risk-adjusting model including demographic information, Charlson-Deyo score, and the number of dysfunctional organ systems for each age stratum. The number of dysfunctional organ systems was categorized as none, one, or two or more, based on preliminary analysis of odds of sepsis-associated death. We developed a final risk-adjusting model using these covariates and adding sepsis-associated co-morbidities that altered the point estimate of the adjusted odds ratio of any of the insurance categories by ≥15% and/or that had a statistically significant association (Wald *P *< 0.05) with sepsis-associated mortality.

We assessed whether the association between lack of health insurance and sepsis-associated mortality was consistent within strata based on hospital sepsis volume or hospital sepsis-associated mortality rates. Hospitals with fewer than 20 sepsis-related admissions were excluded from these analyses. We performed analyses separately within strata based on quartiles of hospital-based sepsis-associated admission rates (that is, the proportion of total admissions involving sepsis) or within strata based on hospital-based sepsis-associated mortality rates (that is, the percentage of sepsis patients dying in the hospital). In each analysis, we combined the middle two quartiles of hospitals to produce three strata of hospitals (for example, the highest quartile of sepsis-associated admission rates, middle quartiles of sepsis-associated admission rates, and lowest quartile of sepsis-associated admission rates). To account for the influence of lack of insurance on sepsis-related mortality due to factors other than the overall performance of the hospital, we ranked hospitals by sepsis-associated mortality rate among patients with insurance (for example, excluding uninsured patients). After stratifying hospitals, subjects without insurance were then included in the datasets for analysis.

We refit the final age-stratum-specific risk-adjusting models developed as described previously for each group, which was now divided both by age group and by hospital strata, defined either by sepsis-associated admission rate or mortality rate. If the association between lack of insurance and sepsis-associated mortality was due to uninsured patients receiving care in hospitals with different rates of sepsis or sepsis-associated mortality, we anticipated that the observed association would be lost once the analyses adjusted for these differences.

To assess any effect of discharge bias due to lack of insurance on sepsis-associated mortality, we re-estimated the odds ratio for uninsured patients, compared to patients with private insurance assuming a mortality rate of 10 to 50% among patients discharged to skilled nursing and intermediate care facilities. Because the sampling strategy was not stratified based on insurance category, we did not risk-adjust these estimates.

All analyses were performed using survey-weight-adjusting procedures in SAS 9.1 (SAS Institute, Inc., Cary, NC, USA). We used two-sided alpha values and considered a *P*-value <0.05 to be statistically significant.

Portions of these analyses were presented in part at the 2008 American Thoracic Society International Conference and summary statistics are included in a systematic review [18].

## Results

### Insurance category and sepsis

In 2003, sepsis was involved in 1 in 35 admissions (2.9%) and consumed 1 in 13 hospital days (7.7%). Of these admissions, 52.7% were associated with organ failure (for example, severe sepsis) and 14.8% had associated shock. Among sepsis patients, 20.6% died during hospitalization, accounting for 1 in 4.3 of all deaths during hospitalization (23.2%).

Tables 1 and 2 show the differences in age, race and co-morbidities between the insurance categories for those 18 to 64 and 65+ years old, respectively. As expected, Medicare was a more common form of insurance among those 65+ and Medicaid alone (1.6%) and Uninsured (0.3%) patients were uncommon among the older age stratum. Sepsis-associated admissions were more frequent among those 65+ (4.3% of hospitalizations) than those 18 to 64 (1.9%). Among patients 18 to 64 years old, patients with Medicare with or without Medicaid had the highest percentage of hospital admissions which were sepsis-associated (4.6% for each insurance category). Among those 65+, the highest percentages of sepsis-associated admissions were observed in those with Medicaid + Medicare (6.4%) and those with Medicaid alone (5.8%). In both age strata, the patients with private insurance had the lowest percentage of sepsis-associated admissions (1.4% in 18 to 64 years and 3.8% in 65+ years), but the observed rates were similar to those in the uninsured patients. As shown in Table 3, sepsis-associated admissions were older, more commonly men, and had higher rates of sepsis-associated co-morbidities.

**Table 1:** Insurance category and covariates of interest, 18 to 64 years old

	Medicaid	Medicare	Medicaid + Medicare	Uninsured	Private insurance
Admissions, 10^3 ^(%)	3,920.6 (23.6%)	1,673.9 (10.1%)	537.0 (3.2%)	1,206.5 (7.3%)	9,260.6 (55.8%)
Age, Mean (95% C.I.)	35.5 (35.2 to 35.9)	51.2 (50.9 to 51.6)	48.5 (48.2 to 48.8)	39.3 (39.1 to 39.5)	42.2 (42.0 to 42.5)
Female, 10^3 ^(% of admissions)	2,975.5 (76.1%)	810.2 (48.5%)	292.4 (54.5%)	586.6 (48.3%)	6,187.8 (67.1%)
Race, 10^3 ^(% of admissions)					
*White*	1,246.8 (31.8%)	792.2 (47.3%)	226.7 (42.2%)	486.6 (39.9%)	4,678.4 (51.5%)
*Black*	735.3 (18.8%)	263.8 (15.8%)	108.8 (20.3%)	196.7 (16.2%)	775.2 (8.4%)
*Hispanic*	812.9 (20.7%)	111.9 (6.7%)	35.7 (6.7%)	180.1 (14.8%)	635.2 (6.9%)
*Asian or Pacific Islander*	73.7 (1.9%)	15.1 (0.9%)	1.7 (0.3%)	17.1 (1.4%)	202.2 (2.2%)
*Native American*	9.6 (0.2%)	2.6 (0.2%)	1.1 (0.2%)	3.1 (0.3%)	13.9 (0.2%)
*Other*	112.9 (2.9%)	20.8 (1.2%)	8.7 (1.6%)	48.5 (4.0%)	202.5 (2.2%)
*Missing*	929.2 (23.7%)	467.5 (27.9%)	154.3 (28.7%)	286.1 (23.5%)	2,663.2 (28.8%)
Quartile of median annual household income by zip code, 10^3 ^(% of admissions)					
*<$36,000*	1,681.4 (43.9%)	553.4 (34.1%)	225.0 (43.0%)	407.0 (34.8%)	1,684.0 (18.6%)
*$36,000 to <$45,000*	1,084.6 (28.3%)	470.7 (29.0%)	142.1 (27.2%)	352.1 (30.1%)	2,176.6 (24.0%)
*$45,000 to <$60,000*	733.3 (19.1%)	360.0 (22.2%)	102.7 (19.6%)	258.5 (22.1%)	2,549.3 (28.1%)
*≥$60,000*	332.4 (8.7%)	239.4 (14.7%)	53.1 (10.2%)	152.7 (13.1%)	2,666.5 (29.4%)
Sepsis-associated conditions, 10^3 ^(% of admissions)					
*Chronic liver disease*	81.8 (2.1%)	48.5 (2.9%)	14.9 (2.8%)	26.4 (2.2%)	90.5 (1.0%)
*Hematologic malignancy*	28.5 (0.7%)	21.0 (1.3%)	4.4 (0.8%)	5.9 (1.1%)	105.0 (0.5%)
*Non-hematologic malignancy*	129.5 (3.3%)	77.1 (4.6%)	18.3 (3.4%)	31.5 (2.6%)	522.5 (5.6%)
*End-stage renal disease*	17.5 (0.4%)	44.4 (2.7%)	12.8 (2.4%)	2.6 (0.3%)	24.1 (0.2%)
*HIV*	59.7 (1.5%)	27.5 (1.6%)	10.5 (2.0%)	11.0 (0.9%)	24.3 (0.3%)
*Alcohol dependence*	134.1 (3.4%)	53.6 (3.2%)	18.6 (3.5%)	87.1 (7.2%)	165.0 (1.8%)
*Organ transplantation*	13.4 (0.3%)	67.2 (4.0%)	11.5 (2.1%)	1.4 (0.1%)	51.6 (0.6%)
*Infection due to device*	9.2 (0.2%)	14.0 (0.8%)	4.0 (0.8%)	1.7 (0.1%)	22.7 (0.2%)
*Red blood cell transfusion*	130.3 (3.3%)	93.6 (5.6%)	30.1 (5.6%)	41.2 (3.4%)	304.3 (3.3%)
Co-morbidity index (Charlson-Deyo) categories, 10^3 ^(% of admissions)					
*0 points*	2,650.5 (67.6%)	588.4 (35.1%)	198.5 (37.0%)	818.2 (69.5%)	6,439.0 (67.2%)
*1 point*	631.0 (16.1%)	451.4 (27.0%)	148.0 (27.6%)	248.4 (16.3%)	1,508.2 (20.4%)
*2 to 8 points*	616.8 (15.7%)	617.5 (36.9%)	186.1 (34.6%)	147.3 (13.6%)	1,259.9 (12.1%)
*9 or more points*	22.3 (0.6%)	16.6 (1.0%)	4.5 (0.8%)	4.3 (0.6%)	53.5 (0.4%)
Sepsis-associated admissions, 10^3^					
*Sepsis (% of admissions)*	75.3 (1.9%)	76.2 (4.6%)	25.0 (4.6%)	18.8 (1.5%)	127.3 (1.4%)
*Severe sepsis (% of sepsis admissions) *	41.0 (54.5%)	47.3 (62.1%)	15.5 (62.0%)	9.2 (48.8%)	58.1 (45.6%)
*Septic shock (% of sepsis admissions)*	10.6 (14.1%)	11.1 (14.6%)	3.4 (13.8%)	2.7 (14.6%)	17.7 (13.9%)

The study cohort was divided into age strata based on age qualification for Medicare. Insurance categories were determined based on primary and secondary payers identified by the data source (see *Methods*). Numbers represent totals from the full weighted sample and are presented as factors of 10^3^. Percentages are based on insurance category by age stratum (unless otherwise indicated). Categorization of co-morbidity index was based upon preliminary analyses examining best fit with odds of sepsis. Severe sepsis and septic shock was categorized as a sepsis-associated admission with ICD-9 codes for any organ failure or for shock, respectively.

**Table 2:** Insurance category and covariates of interest, 65+ years old

	Medicaid	Medicare alone	Medicaid + Medicare	Uninsured	Private insurance plus Medicare
Admissions, 10^3 ^(%)	197.7 (1.6%)	6,696.1 (54.8%)	921.8 (7.6%)	39.4 (0.3%)	4,355.1 (35.7%)
Age, Mean (95% C.I.)	75.1 (74.5 to 75.7)	77.9 (77.7 to 78.0)	77.7 (77.6 to 77.9)	75.2 (74.8 to 75.7)	77.8 (77.6 to 77.9)
Female, 10^3 ^(% of admissions)	131.4 (66.6%)	3,844.0 (57.4%)	655.1 (71.1%)	22.5 (56.4%)	2,463.3 (56.6%)
Race, 10^3 ^(% of admissions)					
*White*	47.2 (24.0%)	4,020.8 (60.0%)	374.5 (40.6%)	14.6 (36.6%)	2,579.2 (59.2%)
*Black*	24.8 (12.5%)	469.5 (7.0%)	164.9 (17.9%)	4.8 (12.1%)	182.4 (4.2%)
*Hispanic*	59.3 (30.0%)	417.7 (6.2%)	113.7 (12.3%)	9.6 (24.1%)	104.9 (2.4%)
*Asian or Pacific Islander*	20.7 (10.5%)	133.4 (2.0%)	10.1 (1.1%)	2.0 (4.9%)	19.4 (0.4%)
*Native American*	0.9 (0.5%)	5.3 (0.1%)	1.9 (0.2%)	0.2 (0.5%)	2.3 (0.1%)
*Other*	10.0 (5.1%)	70.5 (1.1%)	25.8 (2.8%)	2.9 (7.2%)	66.5 (1.5%)
*Missing*	34.7 (17.5%)	1,578.8 (23.6%)	231.0 (25.1%)	5.8 (14.6%)	1,400.4 (32.2%)
Quartile of median annual household income by zip code, 10^3 ^(% of admissions)					
*<$36,000*	75.7 (38.8%)	1,734.4 (26.5%)	433.9 (48.2%)	10.5 (28.0%)	952.2 (22.3%)
*$36,000 to <$45,000*	52.7 (27.0%)	1,848.2 (28.3%)	217.7 (24.2%)	10.4 (27.9%)	1,116.7 (26.1%)
*$45,000 to <$60,000*	38.2 (19.6%)	1,640.7 (25.1%)	159.3 (17.7%)	8.7 (23.2%)	1,172.8 (27.4%)
*≥$60,000*	28.6 (14.6%)	1,318.2 (20.2%)	89.8 (10.0%)	7.8 (20.9%)	1,037.1 (24.2%)
Sepsis-associated conditions, 10^3 ^(% of admissions)					
*Chronic liver disease*	4.4 (2.2%)	76.8 (1.1%)	11.8 (1.3%)	0.7 (1.7%)	42.1 (1.0%)
*Hematologic malignancy*	2.5 (1.2%)	124.2 (1.9%)	10.4 (1.1%)	0.4 (1.1%)	90.1 (2.1%)
*Non-hematologic malignancy*	17.6 (8.9%)	610.0 (9.1%)	58.5 (6.3%)	4.3 (10.8%)	415.4 (9.5%)
*End-stage renal disease*	3.6 (1.8%)	95.1 (1.4%)	17.2 (1.9%)	0.4 (0.9%)	56.6 (1.3%)
*HIV*	0.3 (0.2%)	1.9 (0.03%)	0.4 (0.05%)	0.01 (0.02%)	0.4 (0.01%)
*Alcohol dependence*	1.8 (0.9%)	51.9 (0.8%)	7.3 (0.8%)	0.5 (1.3%)	22.8 (0.5%)
*Organ transplantation*	0.3 (0.2%)	16.0 (0.2%)	1.3 (0.1%)	0.04 (0.1%)	9.4 (0.2%)
*Infection due to device*	0.7 (0.3%)	30.6 (0.5%)	4.3 (0.5%)	0.07 (0.2%)	20.1 (0.5%)
*Red blood cell transfusion*	17.1 (8.7%)	538.0 (8.0%)	87.5 (9.5%)	2.6 (6.5%)	382.9 (8.8%)
Co-morbidity index (Charlson-Deyo) categories, 10^3 ^(% of admissions)					
*0 points*	49.7 (25.1%)	1,853.2 (27.7%)	192.9 (20.9%)	14.0 (35.0%)	1,301.8 (29.9%)
*1 point*	60.3 (30.5%)	2,034.6 (30.4%)	288.6 (31.3%)	11.9 (29.7%)	1,313.7 (30.2%)
*2 to 8 points*	84.8 (42.9%)	2,703.2 (40.4%)	428.6 (46.5%)	13.4 (33.6%)	1,669.3 (38.3%)
*9 or more points*	3.0 (1.5%)	105.1 (1.6%)	11.8 (1.3%)	0.7 (1.6%)	70.4 (1.6%)
Sepsis-associated admissions, 10^3^					
*Sepsis (% of admissions)*	11.4 (5.8%)	288.5 (4.3%)	58.7 (6.4%)	1.5 (3.8%)	163.2 (3.8%)
*Severe sepsis (% of sepsis admissions) *	7.2 (62.6%)	154.7 (53.6%)	30.4 (51.8%)	0.9 (60.8%)	81.6 (50.0%)
*Septic shock (% of sepsis admissions)*	2.2 (19.0%)	44.5 (15.4%)	8.8 (15.0%)	0.3 (20.3%)	24.1 (14.8%)

The study cohort was divided into age strata based on age qualification for Medicare. Insurance categories were determined based on primary and secondary payers identified by the data source (see *Methods*). Numbers represent totals from the full weighted sample and are presented as factors of 10^3^. Percentages are based on insurance category by age stratum (unless otherwise indicated). Categorization of co-morbidity index was based upon preliminary analyses examining best fit with odds of sepsis. Severe sepsis and septic shock was categorized as a sepsis-associated admission with ICD-9 codes for any organ failure or for shock, respectively.

**Table 3:** Sepsis and covariates of interest

	No sepsis	Sepsis	Total
Admissions, 10^6 ^(%)	27.98 (97.1%)	0.85 (2.9%)	29.84
Age, Mean (95% C.I.)	56.6 (56.2 to 57.0)	67.3 (66.9 to 67.7)	56.5 (56.1 to 56.9)
Female, 10^6 ^(% of admissions)	17.52 (62.8%)	0.45 (52.8%)	17.96 (62.5%)
Race, 10^6 ^(% of admissions)			
*White*	14.14 (50.5%)	0.42 (49.5%)	14.56 (50.5%)
*Black*	2.81 (10.0%)	0.11 (13.4%)	2.93 (10.2%)
*Hispanic*	2.41 (8.6%)	0.07 (8.2%)	2.48 (8.6%)
*Asian or Pacific Islander*	0.48 (1.7%)	0.02 (2.2%)	0.50 (1.7%)
*Native American*	0.04 (0.1%)	0.001 (0.2%)	0.04 (0.1%)
*Other*	0.56 (2.0%)	0.01 (1.6%)	0.57 (2.0%)
*Missing*	7.54 (27.0%)	0.21 (24.8%)	7.75 (26.9%)
Quartile of median annual household income by zip code, 10^6 ^(% of admissions)			
*<$36,000*	7.51 (27.5%)	0.25 (30.0%)	7.76 (27.5%)
*$36,000 to <$45,000*	7.26 (26.5%)	0.22 (26.1%)	7.47 (26.5%)
*$45,000 to <$60,000)*	6.83 (25.0%)	0.20 (24.0%)	7.02 (24.9%)
*≥$60,000*	5.76 (21.1%)	0.16 (19.9%)	5.93 (21.0%)
Sepsis-associated co-morbidities, 10^3 ^(% of admissions)			
*Chronic liver disease*	367.3 (1.3%)	30.5 (3.6%)	397.8 (1.4%)
*Hematologic malignancy*	350.4 (1.3%)	42.0 (5.0%)	392.4 (1.4%)
*Non-hematologic malignancy*	1,800.5 (6.4%)	84.2 (9.9%)	1,884.7 (6.5%)
*End-stage renal disease*	244.8 (0.9%)	29.3 (3.5%)	274.1 (1.0%)
*HIV*	123.1 (0.4%)	13.0 (1.5%)	136.1 (0.5%)
*Alcohol dependence*	528.4 (1.9%)	14.3 (1.7%)	542.8 (1.9%)
*Organ transplantation*	157.4(0.6%)	14.8 (1.8%)	172.3 (0.6%)
*Infection due to device*	88.6 (0.3%)	18.7 (2.2%)	107.4 (0.4%)
*Red blood cell transfusion*	1,484.3 (5.3%)	143.2 (16.9%)	1,627.5 (5.6%)
Co-morbidity index (Charlson-Deyo) categories, 10^6 ^(% of admissions)			
*0 points*	13.89 (49.6%)	0.22 (26.0%)	14.11 (48.9%)
*1 point*	6.47 (23.1%)	0.22 (26.4%)	6.70 (23.2%)
*2 to 8 points*	7.34(26.2%)	0.39 (45.7%)	7.73 (26.8%)
*9 or more points*	0.28(1.0%)	0.02 (1.9%)	0.29 (1.0%)
Number of organ failures, 10^6 ^(% of admissions)			
*None*	24.63 (88.1%)	0.40(47.3%)	25.04 (86.9%)
*One*	2.75 (9.8%)	0.25 (29.6%)	3.00 (10.4%)
*Two or more*	0.59 (2.1%)	0.20 (23.1%)	0.78 (2.7%)

Numbers represent totals from the full weighted sample. Numbers are presented as factors of 10^3 ^or 10^6 ^as indicated. Categorization of co-morbidity index was based upon preliminary analyses examining best fit with odds of sepsis.

Table 4 displays the age-strata-specific unadjusted and risk-adjusted association between insurance group and sepsis-associated hospitalization. In the younger age stratum, when compared to those with private insurance, all other insurance groups had significantly higher unadjusted and adjusted odds of a sepsis-associated hospitalization. The increase in risk-adjusted odds were most pronounced among those with Medicaid + Medicare (adjusted odds ratio 2.22, *P *< 0.0001) and among those with Medicare (AOR 1.96, *P *< 0.0001). In the older age stratum, those with Medicaid + Medicare (AOR 1.62, *P *< 0.0001), Medicaid alone (AOR 1.43, *P *< 0.0001) and Medicare alone (AOR 1.13, *P *< 0.0001) had significantly higher risk-adjusted odds of a sepsis-associated hospitalization than those with private insurance plus Medicare. Uninsured patients had risk-adjusted odds of sepsis-associated hospitalization similar to that seen in the reference group.

**Table 4:** Unadjusted and risk-adjusted association between insurance category and sepsis-associated hospitalization by age strata

	Unadjusted	*P-*value	Adjusted	*P-*value
**18 to 64 years**				
*Medicaid*	1.40 (1.34 to 1.48)	<0.0001	1.50 (1.44 to 1.56)	<0.0001
*Medicare*	3.42 (3.25 to 3.61)	<0.0001	1.96 (1.86 to 2.03)	<0.0001
*Medicaid + Medicare*	3.50 (3.26 to 3.75)	<0.0001	2.22 (2.10 to 2.35)	<0.0001
*Uninsured*	1.13 (1.06 to 1.20)	0.0003	1.18 (1.12 to 1.24)	<0.0001
*Private insurance*	Reference		Reference	
**65+ years**				
*Medicaid*	1.58 (1.44 to 1.73)	<0.0001	1.43 (1.32 to 1.55)	<0.0001
*Medicare alone*	1.16 (1.10 to 1.22)	<0.0001	1.13 (1.08 to 1.19)	<0.0001
*Medicaid + Medicare*	1.75 (1.64 to 1.86)	<0.0001	1.62 (1.54 to 1.71)	<0.0001
*Uninsured*	1.02 (0.88 to 1.18)	0.82	1.04 (0.90 to 1.20)	0.63
*Private insurance plus Medicare *	Reference		Reference	

The study cohort was split into age strata based on age qualifications for Medicare. The risk-adjusting model included demographic information, sepsis-associated co-morbidities, and categorized Charlson-Deyo score (see Methods for details).

### Insurance category and sepsis-associated mortality

Table 5 shows the age-strata-specific discharge disposition and hospital length of stay by insurance category. In both age strata, uninsured sepsis patients were most likely to die during hospitalization and least likely to be discharged to an intermediate/skilled nursing facility. Hospital length of stay was longest among Medicaid patients in both age strata.

**Table 5:** Discharge disposition and hospital length of stay among sepsis patients by insurance category and age strata

	Discharge disposition, 10^3 ^(%)			Hospital length of stay, Days, Mean (95% C.I.)
	Died	Skilled/intermediate nursing facility	Home	
**18 to 64 years**				
*All*	46.1 (14.4%)	64.1 (20.0%)	210.7 (65.7%)	13.7 (13.3 to 14.1)
*Medicaid*	11.9 (15.8%)	17.5 (23.4%)	45.7 (60.8%)	15.2 (14.7 to 15.8)
*Medicare*	11.2 (14.8%)	20.0 (26.5%)	44.5 (58.8%)	13.4 (13.0 to 1.38)
*Medicaid + Medicare*	3.4 (13.7%)	7.9 (32.2%)	13.3 (54.2%)	13.1 (12.7 to 13.6)
*Uninsured*	3.0 (16.2%)	1.8 (9.5%)	13.9 (74.3%)	12.8 (12.2 to 13.4)
*Private insurance*	16.7 (13.1%)	16.8 (13.3%)	93.2 (73.6%)	13.1 (12.6 to 13.7)
**65+ years**				
*All*	128.1 (24.6%)	202.8 (39.0%)	189.0 (36.4%)	11.4 (11.1 to 11.7)
*Medicaid*	3.1 (26.9%)	4.0 (34.7%)	4.4 (38.4%)	15.2 (14.3 to 16.1)
*Medicare alone*	70.2 (24.4%)	113.3 (39.4%)	103.8 (36.1%)	11.3 (11.0 to 11.5)
*Medicaid +Medicare*	14.8 (25.4%)	27.9 (48.0%)	15.4 (26.5%)	11.6 (10.8 to 12.4)
*Uninsured*	0.5 (32.2%)	0.3 (16.5%)	0.8 (51.3%)	12.1 (10.5 to 13.8)
*Private insurance plus Medicare*	39.6 (24.5%)	57.5 (35.6%)	64.6 (40.0%)	11.2 (10.8 to 11.6)

The study cohort was divided into age strata based on age qualification for Medicare. Insurance categories were determined based on primary and secondary payers identified by the data source (see *Methods*). Insurance categories for admissions among those 18 to 64 years old and 65+ years old were identical except the "Medicare" and "Private" categories. Among those <65 years old, those with Medicare with or without Private insurance were categorized as "Medicare." Those with private insurance without Medicare were then categorized as "Private." Among those 65+ years, only those with Medicare and no secondary payer were categorized as "Medicare." Those with Medicare and private insurance were categorized as "Private Insurance plus Medicare." Numbers represent totals from the full weighted sample and are presented as factors of 10^3^. Percentages are based on insurance category by age stratum. Discharge disposition was categorized from the discharge record (see Methods for details).

The unadjusted and risk-adjusted odds of hospital mortality among sepsis patients are shown in Table 6. Among those 18 to 64 years, there was a significantly higher risk-adjusted odds of sepsis-associated mortality among uninsured patients (AOR 1.45, *P *< 0.0001) and among patients with Medicaid (AOR 1.17, *P *< 0.0001), compared to those with private insurance. An increased unadjusted odds of sepsis-associated mortality among patients with Medicare was not statistically significant after risk adjustment. Among patients 65+ years, uninsured patients had significantly higher risk-adjusted odds of sepsis-associated mortality (AOR 1.45, *P *= 0.0048), compared to the reference group. Those with Medicare alone had significantly lower odds of sepsis-associated mortality (AOR 0.92, *P *= 0.0072) but this association was not statistically significant in the unadjusted analyses (OR 1.00, *P *= 0.91).

**Table 6:** Unadjusted and risk-adjusted association between insurance category and sepsis-associated mortality by age strata

	Unadjusted	*P-*value	Adjusted	*P-*value
**18 to 64 years**				
*Medicaid*	1.24 (1.15 to 1.33)	<0.0001	1.17 (1.08 to 1.26)	<0.0001
*Medicare*	1.14 (1.08 to 1.22)	<0.0001	1.04 (0.97 to 1.12)	0.24
*Medicaid + Medicare*	1.05 (0.95 to 1.15)	0.36	1.07 (0.97 to 1.18)	0.19
*Uninsured*	1.28 (1.16 to 1.41)	<0.0001	1.45 (1.29 to 1.62)	<0.0001
*Private insurance*	Reference		Reference	
**65+ years**				
*Medicaid*	1.13 (0.98 to 1.31)	0.09	0.99 (0.87 to 1.13)	0.90
*Medicare alone*	1.00 (0.94 to 1.06)	0.91	0.92 (0.86 to 0.98)	0.0072
*Medicaid + Medicare*	1.05 (0.99 to 1.12)	0.12	1.06 (1.00 to 1.13)	0.07
*Uninsured*	1.47 (1.15 to 1.86)	0.0018	1.45 (1.12 to 1.89)	0.0048
*Private insurance plus Medicare *	Reference		Reference	

The study cohort was split into age strata based on age qualifications for Medicare. The risk-adjusting model included demographic information, categorized Charlson-Deyo score, number of dysfunctional organ systems, and sepsis-associated co-morbidities which met *a priori *criteria for inclusion, (see Methods for details).

Uninsured sepsis patients who survived hospitalization were significantly less likely to be discharged to an extended care facility than sepsis survivors with private insurance (Table 5). We tested hypothetical mortality rates between 10% and 50% among patients discharged to an extended care facility. For both age strata, the unadjusted odds of sepsis-associated mortality were significantly higher among the uninsured, provided less than 20% of patients discharged to an intermediate/skilled nursing facility were reclassified as having died. The point estimates of the odds ratios for sepsis-related mortality remained higher in the uninsured patients until 30% or more of the patients discharged to an intermediate/skilled nursing facility were reclassified as having died in both age strata.

To explore the possibility that uninsured patients received care in hospitals with less experience in caring for sepsis or with poorer overall outcomes from sepsis, we placed hospitals into groups based on quartiles of either sepsis-associated admission rate (for example, the percentage of admissions involving sepsis) or sepsis-associated mortality rate (for example, the percentage of sepsis patients dying in the hospital), respectively. This resulted in a significant trend over the lowest, middle and highest groups of hospitals based on sepsis-associated admission rates (1.4% vs. 2.5% vs. 4.0% sepsis-associated hospitalizations, respectively, *P *< 0.0001 by Cochrane-Armitage trend test) and based on sepsis-associated mortality rates (12.6% vs. 19.6% vs. 27.6% mortality among sepsis patients, respectively, *P *< 0.0001). There was a significant trend of uninsured admissions to hospitals categorized by sepsis-associated admission rates (4.2% of admissions in lowest quartile, 4.3% in middle quartiles, 4.8% in highest quartile hospitals, *P *< 0.0001 by Cochrane-Armitage trend test) and when categorized by sepsis-associated mortality rates (3.4% of admissions in lowest quartile hospitals, 4.4% in middle quartile hospitals, 4.8% in highest quartile hospitals, *P *< 0.0001 by Cochrane-Armitage trend test). When the risk adjusting regressions were refit to the age-stratum within each hospital group, the odds of sepsis-associated mortality remained elevated for the uninsured patients compared to the reference group (Table 7). In the older age stratum, not all analyses were statistically significant but the point estimate was similar to that found for the overall cohort.

**Table 7:** Sepsis-associated mortality among uninsured patients, stratified by hospital-based rates of sepsis-associated admission or sepsis-associated mortality

Age stratum	Total cohort	Sepsis-associated admission rate			Sepsis-associated mortality rate		
		Admissions to hospitals in lowest quartile	Admissions to hospitals in middle quartiles	Admissions to hospitals in highest quartile	Admissions to hospitals in lowest quartile	Admissions to hospitals in middle quartiles	Admissions to hospitals in highest quartile
18 to 64 years	1.45 (1.29 to 1.62)	3.76 (2.52 to 5.63)	1.36 (1.19 to 1.57)	1.49 (1.21 to 1.83)	1.53 (1.12 to 2.08)	1.53 (1.34 to 1.74)	1.27 (1.04 to 1.54)
65+ years	1.46 (1.12 to 1.89)	8.67 (2.33 to 32.33)	1.37 (0.98 to 1.90)	1.52 (0.96 to 2.40)	1.59 (1.06 to 2.39)	1.33 (0.90 to 1.97)	1.62 (1.11 to 2.36)

Hospitals were divided into categories by percentage of admissions involving sepsis ("sepsis-associated admission rate") and by percentage of sepsis-associated admissions dying in the hospital ("sepsis-associated mortality rate"). Hospitals were divided into quartiles and the middle two quartiles were combined. The adjusted odds ratios (95% confidence intervals) are referent to patients with any private insurance and adjusted for covariates included in the final risk-adjusting age-strata specific models (see Methods for details).

## Discussion

In this retrospective cohort study, the risk-adjusted odds of a sepsis-associated admission were significantly increased among those with Medicare and/or Medicaid in a younger (18 to 64 years) and older (65+ years) age stratum, compared to those with private insurance. Uninsured patients in the younger age stratum also had higher risk-adjusted odds of sepsis-associated hospitalization. We also found consistently increased odds of sepsis-associated mortality among uninsured patients, compared to those with private insurance.

In the United States, insurer is a complex construct determined by age, chronic health conditions, employment status, income level, state of residence and other factors. Barriers to health care that might prevent sepsis (for example, immunizations) are likely to be different among patients with private insurance compared to those with Medicare compared to those with Medicaid compared to those with no insurance. Mechanisms related to social disadvantages related to a lack of private insurance are likely contributors to the observed results, including those resulting from a lack of commercial insurance (for example, less access to care) or leading to a lack of commercial insurance (for example, unemployment). However, we cannot exclude contributions from the actual mechanism of health care reimbursement and its associated benefits (for example, wellness programs). Differences in co-morbidities not currently known to be associated with sepsis, in socioeconomic status, in environmental and genetic factors and provided care, both before, during and after sepsis may be additional factors which could account for some of the observed disparities between insurance categories. Regardless of the mechanism, the finding of a higher unadjusted rate of sepsis-associated admissions, a diagnosis which consumes significant health care resources [1], may be enough of an incentive to prompt greater attention on the care and outcome of Medicare and Medicaid patients with sepsis.

The specific reasons for greater risk-adjusted odds of sepsis-associated hospitalizations among adults without private insurance are uncertain but could include residual confounding by co-morbidities, disability and frailty not fully adjusted in our analyses. We stratified our analyses by age to account for some of the differences in qualifying criteria for certain insurance types (for example, age alone qualifies those 65+ for Medicare while younger patients must be permanently disabled, have end-stage renal disease, and so on). An inability to account for some of these differences (for example, frailty) may be responsible for the difference in magnitude of association between Medicare and sepsis among the younger (AOR 1.96) and the older strata (AOR 1.13). The NIS is abstracted from records of hospitalizations and is not a true population-based database. Therefore, the increased odds of sepsis are most purely interpreted as increased odds of hospitalization with sepsis compared to hospitalization for reasons other than sepsis. An alternate interpretation of our data is that those with private insurance are more likely to be hospitalized for non-sepsis indications, potentially representing a healthy user bias [19], a possibility not excluded in the current analyses. However, the finding of a similar percentage of admissions associated with sepsis among uninsured patients and those with private insurance in the younger (1.5% vs. 1.4% of admissions, respectively) and older age strata (3.8% vs. 3.8% of admissions, respectively) is reassuring. Sepsis could also occur at a similar rate among all patients but patients without private insurance have more severe disease and/or greater access to care, resulting in higher rates of hospitalization.

Uninsured patients had higher risk-adjusted odds of sepsis-associated mortality. Among those 18 to 64 years, patients with Medicaid also had significantly higher risk-adjusted odds of sepsis-associated mortality, but this was of a smaller magnitude than seen for uninsured patients. While unproven by the available data, it is possible that these patients delay care for sepsis. Perhaps supporting such an explanation is the finding of an increased rate of severe sepsis and septic shock among uninsured and Medicaid patients with sepsis compared to those with private insurance. While we adjusted for numbers of organ failures, using administrative data prevented the use of a physiology-based severity of illness system that might provide greater detail regarding this observation. Multiple studies have reported higher risk-adjusted mortality for critically ill patients without insurance [18,20-23]. To our knowledge, this is the first such study specifically examining this association among sepsis patients. Limitations to these findings are our inability to precisely identify the mechanism of increased sepsis-related mortality among the uninsured and to assess the severity, stability and treatment of co-morbidities which could have affected survival among uninsured sepsis patients. It is also possible that in some hospitals, patients initially admitted without insurance may be moved to the Medicaid group to improve hospital reimbursement. If this occurred in a systematic manner (for example, those living for only a short time with sepsis do not have the required paperwork completed and die as uninsured while those living longer receive Medicaid), this could bias the observed results. Among the older age stratum, patients with Medicare had lower risk-adjusted odds of sepsis-associated mortality compared to patients with Medicare and private insurance. The magnitude of this association was relatively small and only found in the risk-adjusted analyses, raising questions about the clinical significance and validity of this finding.

We found that uninsured patients were significantly more likely to receive care in hospitals with higher sepsis-associated admission rates and in hospitals with higher sepsis-related mortality rates. Any hospital-based effect (for example, higher mortality due to poorer care for sepsis patients) would likely affect all sepsis patients at that hospital and, therefore, would be accounted for in the stratification analysis. We found no evidence of such an effect. Our findings do not, however, eliminate the possibility of differences in care for uninsured patients relative to those with private insurance across all hospitals, delays in presentation for treatment of sepsis, and inadequate pre-sepsis treatment of known sepsis risk-factors leading to more severe disease as possible mechanisms for the observed association. Finally, while uninsured patients were less likely to be discharged to intermediate/skilled nursing facilities, more than 20% of patients would have to be reclassified as dying, rather than being discharged to such facilities, to confound the observed findings. While this mortality rate may seem realistic for sepsis survivors discharged to such a facility, this would have to be the mortality rate over a short interval - namely, during the time that a privately insured patient would have remained hospitalized if she were uninsured (on average, less than one day for the older cohort). Also, a similar length of stay among uninsured sepsis patients and those with private insurance argues against a significant discharge bias.

## Limitations

Primary among the limitations of this study is the use of an administrative database that lacks independent validation with the clinical record and relies on billing codes. We cannot, for example, determine if sepsis was the primary reason for admission or was a nosocomial complication. In the case of sepsis-associated mortality, we cannot determine that sepsis was the actual cause of death. Instead, patients could survive sepsis and die of an alternate cause and yet be considered a sepsis-associated death. Because sampling in NIS is not directly based upon the mix of insurance types, there is the possibility of selection bias for certain types of insurance that could make the observed estimates of association less accurate. Bias in the observed results would likely require a systematic miscoding of sepsis and/or sepsis-associated mortality based on insurance category. For example, if those abstracting charts of patients with private insurance were more likely to code for sepsis than those abstracting charts of patients with Medicaid and/or Medicare, the observed results could be biased. For the purposes of assessing the mechanism(s) of the observed associations, the data included in NIS are limited. For example, while quartiles of median income based on zip code have been used as a surrogate for socioeconomic status [24], it remains a crude surrogate for this construct. We excluded records of patients transferred from or to another short-term acute care hospital to reduce the likelihood of transfer bias and double counting individual patients [6]. However, if there was a disparity in the transfer of sepsis versus non-sepsis patients based on insurance, our observed results may suffer from selection bias.

While not the primary focus of the current study, our incidence rates of sepsis should best be interpreted as "treated incidence" [25]. This requires the recognition of sepsis and the hospitalization of the patient so that the record is included in the database. Considering the low rate of recognition of sepsis by clinicians [26], including as a cause of death [27], and the insensitive nature of diagnostic codes for sepsis [8], our reported numbers of sepsis cases may be an underestimate of the true incidence.

## Conclusions

Using a national discharge database, we found higher risk-adjusted odds of a sepsis-associated hospitalization among patients with Medicare and/or Medicaid, compared to those with private insurance. Younger (18 to 64 years) uninsured patients also had higher risk-adjusted odds of sepsis-associated admissions and uninsured sepsis patients in both age strata were more likely to die during hospitalization. While these results provide initial insight into the association between sepsis and insurance category, the specific mechanisms of these associations cannot be definitively determined from the existing data. A prospective, population-based study with longitudinal patient-level information on outpatient care prior to the sepsis admission and inpatient care would allow for a better assessment of additional risk factors for sepsis and sepsis-associated mortality and provide targets for intervention that might mitigate the observed disparities.

## Key messages

• Sepsis is a common reason for hospitalization and is involved in approximately one in four deaths among hospitalized patients.

• Among those under 65 years old, patients without private insurance have significantly higher odds of sepsis-associated hospitalization with the highest odds among those with Medicare with or without Medicaid.

• Among those age 65+, patients with Medicaid with or without Medicare had the highest risk-adjusted odds of sepsis-associated hospitalization.

• Among those with sepsis, uninsured patients have higher odds of hospital mortality compared to those with private insurance.

• Higher sepsis-associated mortality among uninsured patients is not explained by examined demographics, co-morbidities, sepsis-associated organ failures, socioeconomic factors or differences in hospitals.

## Abbreviations

AOR: adjusted odds ratio; CPT: current procedural terminology codes; NIS: nationwide inpatient sample; OR: odds ratio.

## Competing interests

JO is supported by the Davis/Bremer Medical Research Grant and NIH K23 HL075076. The funders had no role in study design, data collection and analysis, decision to publish, or preparation of the manuscript. JO gave a lecture related to sepsis as a result of an unrestricted grant from BRAHMS, Inc. He donated the honorarium to the Sepsis Alliance and received airfare and two nights' hotel accommodations totaling approximately $1,500 (2009).

JO serves on the Board of Directors for the Sepsis Alliance, a not-for-profit organization dedicated to improving awareness and care of septic patients. He is not paid for this directorship.

The other authors all declare that they have no competing interests.

## Authors' contributions

JO contributed to the conception, design, statistical analysis and interpretation of the study and drafting, critical revision, reading and approval of the manuscript. BL contributed to the design, statistical analysis and interpretation of the study and critical revision, reading and approval of the manuscript. NA contributed to the design and interpretation of the study and drafting, critical revision, reading and approval of the manuscript. DL contributed to the conception, design, and interpretation of the study and critical revision, reading and approval of the manuscript. SA contributed to the design and interpretation of the study and drafting, critical revision, reading and approval of the manuscript. SL contributed to the design, statistical analysis and interpretation of the study and critical revision, reading and approval of the manuscript. All authors read and approved the final manuscript.

## References

[B1] Angus DC Linde-Zwirble WT Lidicker J Clermont G Carcillo J Pinsky MR Epidemiology of severe sepsis in the United States: analysis of incidence, outcome, and associated costs of care Crit Care Med 2001 29 1303 1310 10.1097/00003246-200107000-00002 11445675

[B2] McBean M Rajamani S Increasing rates of hospitalization due to septicemia in the US elderly population, 1986-1997 J Infect Dis 2001 183 596 603 10.1086/318526 11170985

[B3] Fronstin P Sources of health insurance and characteristics of the uninsured: analysis of the March 2008 current population survey EBRI Issue Brief 2008 1 33 18811062

[B4] Kumar A Roberts D Wood KE Light B Parrillo JE Sharma S Suppes R Feinstein D Zanotti S Taiberg L Gurka D Kumar A Cheang M Duration of hypotension before initiation of effective antimicrobial therapy is the critical determinant of survival in human septic shock Crit Care Med 2006 34 1589 1596 10.1097/01.CCM.0000217961.75225.E9 16625125

[B5] HCUP Nationwide Inpatient Sample (NIS) 2003 Rockville, MD, Healthcare Cost and Utilization Project (HCUP)

[B6] Westfall JM McGloin J Impact of double counting and transfer bias on estimated rates and outcomes of acute myocardial infarction Med Care 2001 39 459 468 10.1097/00005650-200105000-00006 11317094

[B7] Description of Data Elements Nationwide Inpatient Sample Data Elements Beginning with M-Z 2005 2 Rockville, MD: Healthcare Cost and Utilization Project

[B8] Martin GS Mannino DM Eaton S Moss M The epidemiology of sepsis in the United States from 1979 through 2000 N Engl J Med 2003 348 1546 1554 10.1056/NEJMoa022139 12700374

[B9] O'Brien JM Jr Lu B Ali NA Martin GS Aberegg SK Marsh CB Lemeshow S Douglas IS Alcohol dependence is independently associated with sepsis, septic shock, and hospital mortality among adult intensive care unit patients Crit Care Med 2007 35 345 350 10.1097/01.CCM.0000254340.91644.B2 17205003

[B10] Martin GS Mannino DM Moss M The effect of age on the development and outcome of adult sepsis Crit Care Med 2006 34 15 21 10.1097/01.CCM.0000194535.82812.BA 16374151

[B11] Foreman MG Mannino DM Moss M Cirrhosis as a risk factor for sepsis and death: analysis of the National Hospital Discharge Survey Chest 2003 124 1016 1020 10.1378/chest.124.3.1016 12970032

[B12] Williams MD Braun LA Cooper LM Johnston J Weiss RV Qualy RL Linde-Zwirble W Hospitalized cancer patients with severe sepsis: analysis of incidence, mortality, and associated costs of care Crit Care 2004 8 R291 R298 10.1186/cc2893 15469571 PMC1065011

[B13] Hirschtick RE Glassroth J Jordan MC Wilcosky TC Wallace JM Kvale PA Markowitz N Rosen MJ Mangura BT Hopewell PC Bacterial pneumonia in persons infected with the human immunodeficiency virus. Pulmonary Complications of HIV Infection Study Group N Engl J Med 1995 333 845 851 10.1056/NEJM199509283331305 7651475

[B14] Taylor RW Manganaro L O'Brien J Trottier SJ Parkar N Veremakis C Impact of allogenic packed red blood cell transfusion on nosocomial infection rates in the critically ill patient Crit Care Med 2002 30 2249 2254 10.1097/00003246-200210000-00012 12394952

[B15] Collignon PJ Intravascular catheter associated sepsis: a common problem. The Australian Study on Intravascular Catheter Associated Sepsis Med J Aust 1994 161 374 378 8090116

[B16] Deyo RA Cherkin DC Ciol MA Adapting a clinical comorbidity index for use with ICD-9-CM administrative databases J Clin Epidemiol 1992 45 613 619 10.1016/0895-4356(92)90133-8 1607900

[B17] Houchens R Elixhauser A Final Report on Calculating Nationwide Inpatient Sample (NIS) Variances, 2001 2005 U.S. Agency for Healthcare Research and Quality HCUP Methods Series Report #2003-2

[B18] Fowler RA Noyahr LA Thornton JD Pinto R Kahn JM Adhikari NK Dodek PM Khan NA Kalb T Hill A O'Brien JM Evans D Curtis JR American Thoracic Society Disparities in Healthcare Group An official American Thoracic Society systematic review: the association between health insurance status and access, care delivery, and outcomes for patients who are critically ill Am J Respir Crit Care Med 2010 181 1003 1011 10.1164/rccm.200902-0281ST 20430926 PMC3269233

[B19] MacMahon S Collins R Reliable assessment of the effects of treatment on mortality and major morbidity, II: observational studies Lancet 2001 357 455 462 10.1016/S0140-6736(00)04017-4 11273081

[B20] Danis M Linde-Zwirble WT Astor A Lidicker JR Angus DC How does lack of insurance affect use of intensive care? A population-based study Crit Care Med 2006 34 2043 2048 10.1097/01.CCM.0000227657.75270.C4 16763518

[B21] Horner RD Bennett CL Rodriguez D Weinstein RA Kessler HA Dickinson GM Johnson JL Cohn SE George WL Gilman SC Relationship between procedures and health insurance for critically ill patients with *Pneumocystis carinii *pneumonia Am J Respir Crit Care Med 1995 152 1435 1442 7582274 10.1164/ajrccm.152.5.7582274

[B22] Durairaj L Will JG Torner JC Doebbeling BN Prognostic factors for mortality following interhospital transfers to the medical intensive care unit of a tertiary referral center Crit Care Med 2003 31 1981 1986 10.1097/01.CCM.0000069730.02769.16 12847392

[B23] Schnitzler MA Lambert DL Mundy LM Woodward RS Variations in healthcare measures by insurance status for patients receiving ventilator support Clin Perform Qual Health Care 1998 6 17 22 10177044

[B24] Carlisle DM Leake BD Differences in the effect of patients' socioeconomic status on the use of invasive cardiovascular procedures across health insurance categories Am J Public Health 1998 88 1089 1092 10.2105/AJPH.88.7.1089 9663160 PMC1508262

[B25] Linde-Zwirble WT Angus DC Severe sepsis epidemiology: sampling, selection, and society Crit Care 2004 8 222 226 10.1186/cc2917 15312201 PMC522859

[B26] Poeze M Ramsay G Gerlach H Rubulotta F Levy M An international sepsis survey: a study of doctors' knowledge and perception about sepsis Crit Care 2004 8 R409 R413 10.1186/cc2959 15566585 PMC1065059

[B27] Lakkireddy DR Gowda MS Murray CW Basarakodu KR Vacek JL Death certificate completion: how well are physicians trained and are cardiovascular causes overstated? Am J Med 2004 117 492 498 10.1016/j.amjmed.2004.04.018 15464706

